# Microsatellite‐based analysis reveals *Aedes aegypti* populations in the Kingdom of Saudi Arabia result from colonization by both the ancestral African and the global domestic forms

**DOI:** 10.1111/eva.13661

**Published:** 2024-02-22

**Authors:** Abadi M. Mashlawi, Hussain Alqahtani, Sara A. Abuelmaali, Andrea Gloria‐Soria, Jassada Saingamsook, Martha Kaddumukasa, Ahmad Hassn Ghzwani, Ahmed A. Abdulhaq, Hesham M. Al‐Mekhlafi, Catherine Walton

**Affiliations:** ^1^ Department of Biology, College of Science Jazan University Jazan Kingdom of Saudi Arabia; ^2^ Department of Biology, Faculty of Science University of Tabuk Tabuk Kingdom of Saudi Arabia; ^3^ National Public Health Laboratory Federal Ministry of Health Khartoum Sudan; ^4^ Department of Entomology, Center for Vector Biology & Zoonotic Diseases The Connecticut Agricultural Experiment Station New Haven Connecticut USA; ^5^ Center of Insect Vector Study, Department of Parasitology, Faculty of Medicine Chiang Mai University Chiang Mai Thailand; ^6^ Department of Biological Sciences, Faculty of Science Kyambogo University Kampala Uganda; ^7^ Medical Research Centre Jazan University Jazan Kingdom of Saudi Arabia; ^8^ Department of Medical Laboratory Technology, Faculty of Applied Medical Sciences Jazan University Jazan Kingdom of Saudi Arabia; ^9^ Department of Parasitology, Faculty of Medicine Universiti Malaya Kuala Lumpur Malaysia; ^10^ Department of Parasitology, Faculty of Medicine and Health Sciences Sana'a University Sana'a Yemen; ^11^ Department of Earth and Environmental Sciences, Faculty of Science and Engineering University of Manchester Manchester UK

**Keywords:** *Aedes aegypti*, *Aedes formosus*, Arabian Peninsula, genetic diversity, microsatellites, mosquitoes, population genetics, Saudi Arabia

## Abstract

*The Aedes aegypti* (Linnaeus, 1762) mosquito is the main vector of dengue, chikungunya and Zika and is well established today all over the world. The species comprises two forms: the ancestral form found throughout Africa and a global domestic form that spread to the rest of the tropics and subtropics. In Saudi Arabia, *A. aegypti* has been known in the southwest since 1956, and previous genetic studies clustered *A. aegypti* from Saudi Arabia with the global domestic form. The purpose of this study was to assess the genetic structure of *A. aegypti* in Saudi Arabia and determine their geographic origin. Genetic data for 17 microsatellites were collected for *A. aegypti* ranging from the southwestern highlands of Saudi Arabia on the border of Yemen to the north‐west in Madinah region as well as from Thailand and Uganda populations (as representatives of the ancestral African and global domestic forms, respectively). The low but significant level of genetic structuring in Saudi Arabia was consistent with long‐distance dispersal capability possibly through road connectivity and human activities, that is, passive dispersal. There are two main genetic groupings in Saudi Arabia, one of which clusters with the Ugandan population and the other with the Thailand population with many Saudi Arabian individuals having mixed ancestry. The hypothesis of genetic admixture of the ancestral African and global domestic forms in Saudi Arabia was supported by approximate Bayesian computational analyses. The extent of admixture varied across Saudi Arabia. African ancestry was highest in the highland area of the Jazan region followed by the lowland Jazan and Sahil regions. Conversely, the western (Makkah, Jeddah and Madinah) and Najran populations corresponded to the global domesticated form. Given potential differences between the forms in transmission capability, ecology and behaviour, the findings here should be taken into account in vector control efforts in Saudi Arabia.

## 1 INTRODUCTION

*Aedes aegypt*i (Diptera: Culicidae) is the primary vector of pathogens responsible for yellow fever, dengue, chikungunya and Zika (WHO, 2022). In Saudi Arabia, dengue fever cases have been reported in the country's southwestern regions, that is, Jeddah, Makkah, Madinah, Jazan and Sahil (Al‐Azraqi et al., 2013; Altassan et al., 2019; Fakeeh & Zaki, 2003). Two cases of chikungunya were reported: one qRT‐PCR‐confirmed autochthonous case in Jeddah in 2011 and another IgG‐seropositive case in Jazan in 2021 (Hakami et al., 2021; Hussain et al., 2013). However, there have been no reported cases of Zika to date. In 2013, the incidence rate of dengue cases increased to 21.71 per 100,000 persons/year and dropped to 10.03 in 2021 (Alhaeli et al., 2016; Health, 2019, 2021). In the Jazan region of southwestern Saudia Arabia, the total number of dengue cases was 4985 between 2005 and 2021, but there have been no reported cases in the nearby highland area (Health, 2021). A good understanding of population genetic structure, ecology as well as the genetic make‐up of local *A. aegypti* is essential for understanding the temporal epidemiology and risk of arboviral diseases.

The southwestern islands of the Indian Ocean host the oldest *A. aegypti* populations and are the likely source of *A. aegypti* that colonized Africa and subsequently spread to the rest of the world (Soghigian et al., 2020). *Aedes aegypti* has been in Africa for ~85,000 years, where it accumulated genetic diversity (Soghigian et al., 2020). However, the spread of *A. aegypti* to the rest of the tropics is recent and characterized by a genetic bottleneck resulting in low genetic diversity (Powell & Tabachnick, 2013). The slave trade from Africa to the New World between 1500 and 1650 and commercial shipping between countries are responsible for the spread of *A. aegypti* from Africa (Powell et al., 2018). In the Arabian Peninsula, *A. aegypti* has been present since at least 1956 in Jeddah, Makkah and Jazan, Saudi Arabia (Mattingly & Knight, 1956). Today, *A. aegypti* is found in the south and southwest regions of Saudi Arabia, along the Red Sea (Alikhan et al., 2014), and recently was recorded in areas of the north and central regions of the country (Al‐Rashidi, pers. comm.). The origin of the Saudi Arabian population is unknown.

There are widely considered to be two forms of *A. aegypti*: a dark forest‐dwelling form *A. aegypti formosus (Aaf*) from sub‐Saharan Africa: and the paler coloured domestic form *A. aegypti aegypti* (*Aaa*) that dispersed from West Africa using global trade routes, to the rest of the tropics and subtropics (Powell et al., 2018; Powell & Tabachnick, 2013; Rose et al., 2023). *Aedes aegypti formosus* is generally considered not to occur outside of Africa. In this respect, it is interesting to note that two forms of *A. aegypti*, a dark form and a pale form, have been reported in the Arabian Peninsula (Mattingly & Knight, 1956). We, therefore, hypothesize that *A. aegypti* in Saudi Arabia arises from two sources, the diaspora of the global domestic form and the direct migration of the *A. aegypti formosus* form from Africa. Direct migration from Africa seems highly plausible given that the southwest region of Saudi Arabia up to and including Makkah and Jeddah is considered part of the Afrotropical zoogeographic region (Holt et al., 2013; Lane & Crosskey, 2012). Further, there has been extensive historical trade over the last two to three thousand years using sea routes connecting the Arabian Peninsula and Africa, that primarily used Aden, a Yemeni port (Martin & Vigne, 1997). This provides a viable transportation route for *A. aegypti formosus* from Africa to Saudi Arabia. The presence of *A. aegypti formosus* in Saudi Arabia has been suggested previously (El‐Badry & Al Ali, 2010), and the argument put forward that divergent mtDNA clades in Saudi Arabia support this (Khater et al., 2021). However, the presence of both these clades throughout Africa as well as other tropical countries (Bennett et al., 2016) means such conclusions cannot be drawn from mtDNA.

The Kingdom of Saudi Arabia has experienced rapid urbanization, with a fourfold increase in urbanization from 1950 (21%) to 2015 (83%) (Alahmadi & Atkinson, 2019). Makkah and Jeddah are highly urbanized cities compared to Jazan, Sahil, Najran and Madinah. Unplanned urbanization is associated with the spread of *A. aegypti* and the diseases it transmits (Kolimenakis et al., 2021). In addition, the widespread use of air‐conditioning systems, the water containers of which provide common breeding sites for *A. aegypti*, results in a high abundance of adult *A. aegypti* inside houses (Ali et al., 2021; Khater et al., 2021). *A. aegypti* is abundant year‐round with density reported to peak in April in Madinah (El‐Badry & Al Ali, 2010), January to March in Makkah (Aziz et al., 2012), December and January in Jeddah (Mahyoub, 2015), and November and December in Jazan (Alahmed et al., 2010).

Landscape features such as highways, rivers and primary roads play an important role in the dispersal of *A. aegypti* (Regilme et al., 2021). Passive dispersal through human transportation (likely in the form of immature stages or eggs) has been reported in *A. aegypti* from Argentina (Maffey et al., 2020, 2022), Sri Lanka (Fernando et al., 2020) and Southeast Asia (Hlaing et al., 2010; Huber et al., 2002). Due to the short flight distance (average lifetime dispersal <200 m), active dispersal in *A. aegypti* is unlikely to shape genetic structure at the countrywide level (Moore & Brown, 2022). Passive dispersal was found to increase in urban areas (Maffey et al., 2022) and was weak in mountains or isolated geographical locations (Fernando et al., 2020). This potential for long‐range dispersal constitutes a major threat, as undesired traits such as insecticide resistance or a pathogen could be introduced to new locations.

Microsatellite markers have been widely used to characterize genetic population structure in *A. aegypti* worldwide (Gloria‐Soria et al., 2016). In Saudi Arabia, only two studies from Madinah and Jeddah have addressed the population dynamics of *A. aegypti* using the mitochondrial cytochrome‐c‐oxidase subunit‐1 (CO1) and dehydrogenase subunit‐4 (ND4) (Ali et al., 2016; Khater et al., 2021) gene regions, but no finer scale genetic studies (i.e., using microsatellites or SNPs) have been conducted yet. Similarly, a study of the gene flow dynamics in southern and southwestern Saudi Arabia (i.e., Jazan and Sahil) is still absent (Mashlawi et al., 2022). Understanding population connectivity in this species is critical for determining the efficacy of innovative mosquito control strategies, that is, genetically modified mosquitoes and *Wolbachia* releases. Besides, understanding the genetic composition of *A. aegypti* populations in Saudi Arabia is also important since the two forms may differ in disease risk (Dickson et al., 2014; Weetman et al., 2018). Within this context, in this study, we aim to assess the genetic structure, gene flow regime and number of *A. aegypti* genetic clusters present in Saudi Arabia and test the hypothesis that *A. aegypti* in Saudi Arabia has been formed by admixture between ancestral populations from Africa and the global domestic form.

## 2 MATERIALS AND METHODS

### 2.1 Study sites

**FIGURE 1:**
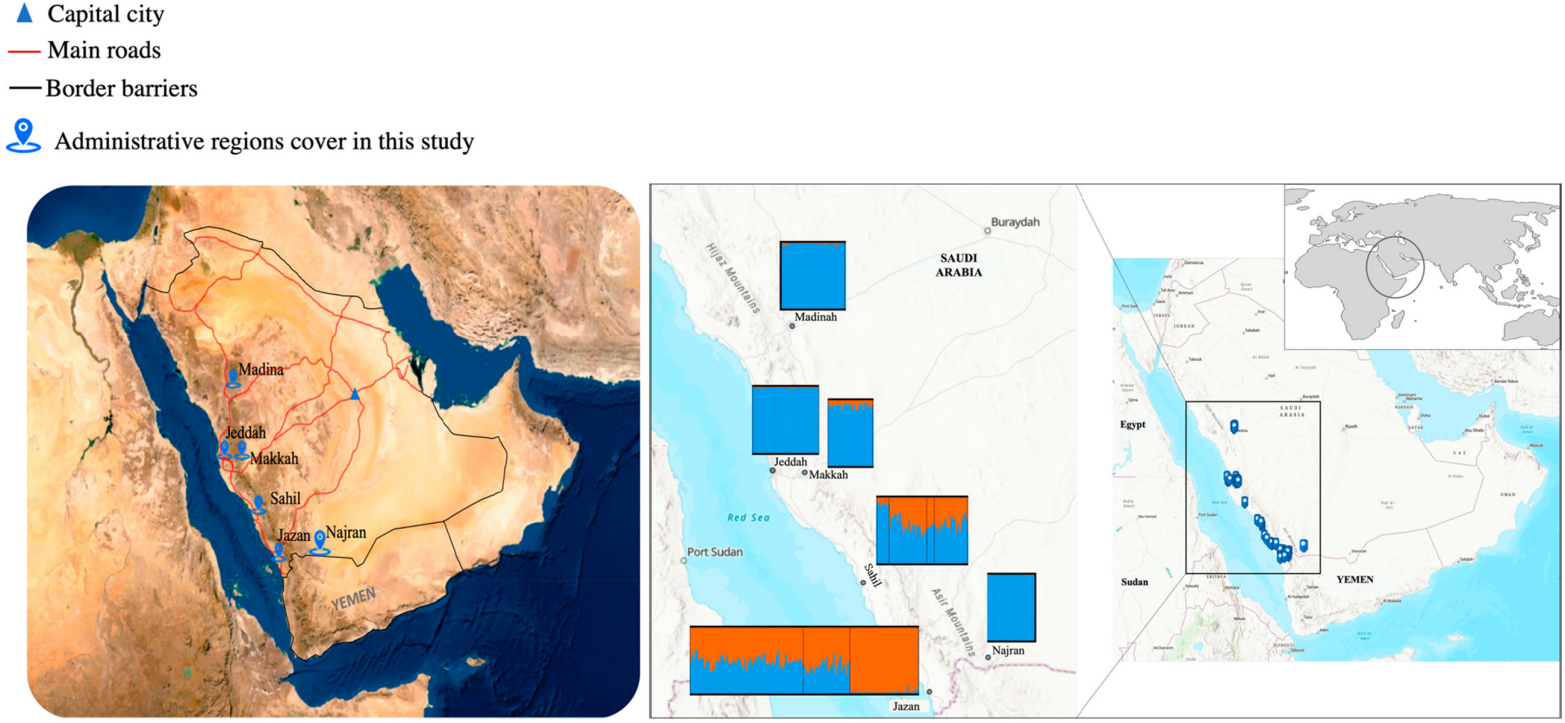
Map of Saudi Arabia showing the sampling locations of *Aedes aegypti*, that is, Jazan, Sahil, Makkah, Jeddah, Madinah and Najran with main road connectivity (left) and the STRUCTURE bars of each population based on *K* = 2 in STRUCTURE software (Pritchard et al., 2000). Colours within each bar represent each genetic cluster, and the percentage of the colour indicates the percentage of ancestry of each cluster for a particular individual in this study (right). The website ArcGIS (https://www.arcgis.com/index.html) was used to generate the map.

Saudi Arabia, which lies in southwestern Asia, is the largest country in the Arabian Peninsula. Geographically, Saudi Arabia is divided into three distinct zones: the rain‐fed highlands of the western and southwestern regions (Sarawat Mountains), the arid and extra‐arid lands of the interior (Najd), and the coastal plain along the Red Sea in the west of Saudi Arabia (known as the Tihamah) that includes the east of the Hejaz and the Asir mountain range (Figure 1).

Most collections for this study came from the Tihamah and Hejaz regions. Six different regions, namely, Jeddah, Makkah, Madinah, Jazan, Sahil and Najran, were included in this study. More information about each region is referred to in Mashlawi et al. (2022). We refer to Makkah, Jeddah and Madinah as ‘western’ in this study. The Sahil region (stretching ~400 km along the Red Sea coast, linking Jazan with Makkah and Jeddah) was used to evaluate spatial connectivity, as it represents a major concern in terms of passive dispersal due to the high level of use of the international road from Yemen to Makkah through Jazan and Sahil. The Najran region (17°32′ N 44°13′ E) also shares a border with Yemen to the south and is known for an ancient Christian settlement around the 7th century (Frankfurter, 1998), which means there has been a long‐term movement of people into this region of Saudi Arabia. To investigate the evolutionary history and test the hypothesis of genetic admixture within Saudi Arabia of the ancestral African and pantropical global forms, population samples from Thailand and Uganda were also included to represent these potential source populations (Table 1). Use of a single population to represent each form is reasonable given that there is far greater genetic differentiation between the forms than there is between populations within the forms (Elnour et al., 2022; Gloria‐Soria et al., 2016).

**TABLE 1:** Country, collection regions, site names (code), number of samples, geographical coordinates and collection date of *Aedes aegypti* used in the present study.

Country	Collection regions	Site names	No. of samples	Geographical coordinates	Collection date
Saudi Arabia	Madinah	Madinah	42	N 24°28′12.4″	2021
				E 39°37′45.1″	
	Makkah	Jeddah	39	N 21°26′48.04″	2020/21
				E 39°11′55.01″	
	Makkah	Makkah	30	N 21°28′48.7″	2020/21
				E 39°47′45.6″	
	Makkah	SahilW	8	N 20°09′07.11″	2020/21
				E 40°16′44.11″	
	Makkah	SahilM	25	N 18°58′16.99″	2020/21
				E 41°19′21.25″	
	Aseer	SahilE	5	N 18°12′51.29″	2020/21
				E 41°32′01.80″	
	Aseer	SahilAs	22	N 17°43′35.86″	2020/21
				E 42°01′45.67″	
	Jazan	Jazan villages	72	N 17°14′23.19″	2019–21
				E 42°36′08.66″	
	Jazan	Jazan urban lowland	16	N 16°55′56.70″	2020/21
				E 42°33′47.74″	
	Jazan	Jazan highland	46	N 17°16′12.08″	2020/21
				E 43°07′25.09″	
	Najran	Najran	32	N 17°29′40.639″	2022
				E 44°08′16.001″	
Thailand	Chiang Mai	Thailand	22	N 18°47′15.8″	2017/18
				E 98°59′03.3″	
Uganda	Zika forest	Uganda	30	N 0°07′17.1″	2020
				E 32°31′34.6″	

### 2.2 Mosquito collections

**FIGURE 2:**
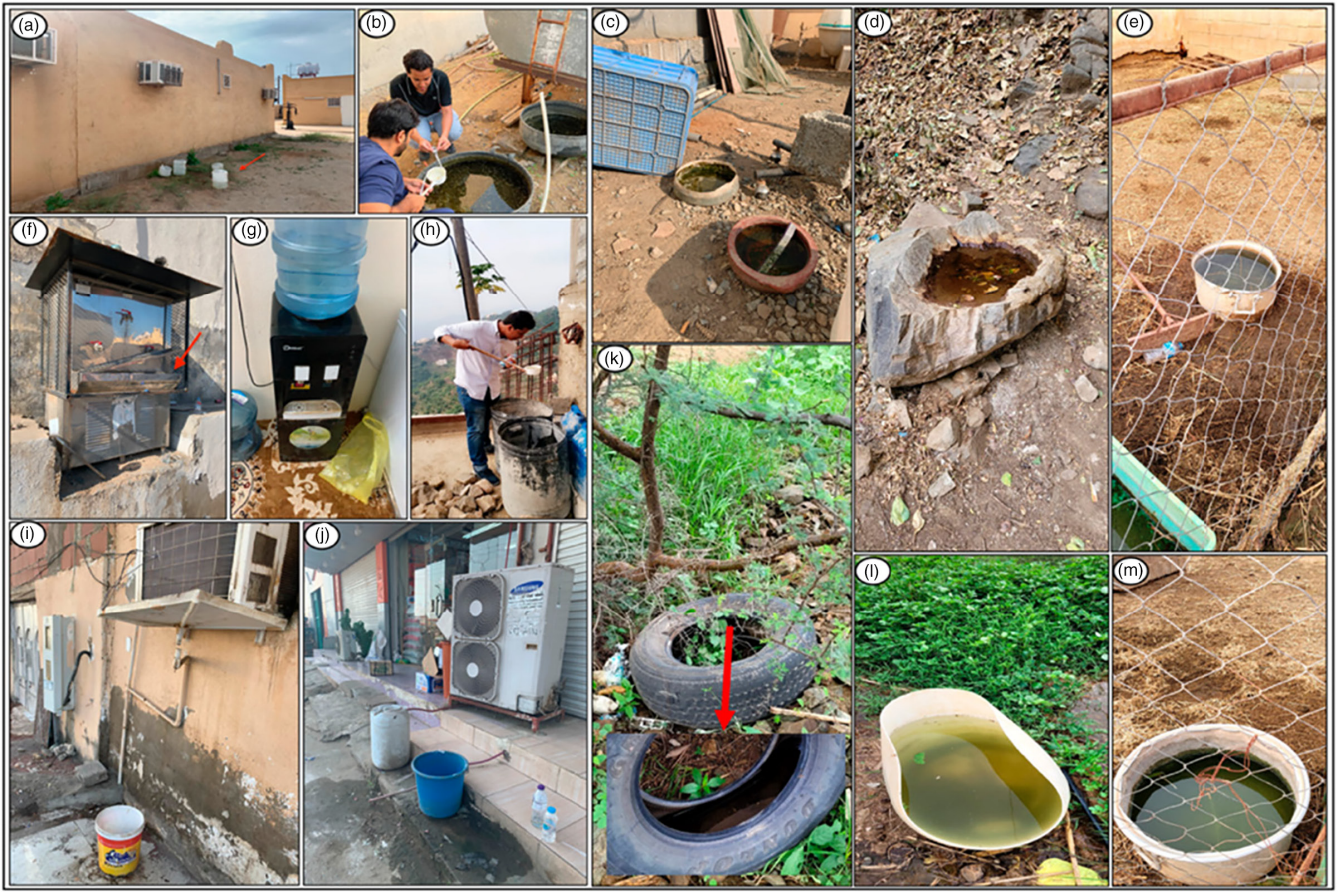
Field photos and examples of potential larval habitat for *Aedes aegypti* in Saudi Arabia. (a, i, j) Air‐conditioning water containers, (b, c, e, m) animals drinking containers, (d) rainwater in rock hole, (f) water coolers in mosques and neighbourhoods, (g) water coolers inside houses, (h) construction containers, (k) discarded car tire and (l) disposable plastic container.

Samples of *A. aegypti* eggs, larvae and pupae were collected from six regions (four administrative areas) in Saudi Arabia between 2019 and 2022 during the wet season (November to February). The samples were collected from different sites including air‐conditioning water containers and disposable plastic containers (Figure 2). The Thailand samples were collected in 2017/18, and the samples from Uganda were collected in 2020.

The samples were collected from 11 locations in Saudi Arabia which were a mix of urban, semi‐rural and rural areas (villages) (Table 1). The maximum distance between sampling locations within a location ranged from 22 to 58 km. Mosquitoes were sampled using a larval dipper from a total of 103 collection sites (detailed in Table 1 and Figure 1). All immature stages were maintained at the Centre for Disease Control and Prevention, Ministry of Health, Sabya, at a temperature of 28 ± 2°C and relative humidity of 75 ± 10%, as previously described (Mashlawi et al., 2020). Collected samples were identified morphologically as *A. aeg*ypti using a mosquito taxonomic key (Rueda, 2004). During this process, the presence of two forms of *A. aegypti* (paler and dark) in Saudi Arabia was noted as previously reported by Mattingly and Knight (1956) (Additional file 1: Figure S1). The dark form was only found in the Jazan highland region. The samples were preserved in tubes with silica gel and transferred to the University of Manchester, UK, for molecular work.

### 2.3 DNA extraction and microsatellite genotyping of *A. aegypti* populations

Genomic DNA was extracted using the DNeasy Blood and Tissue Kit (QIAGEN Sciences, Germantown, MD, USA). As full siblings are expected in larval containers (Schmidt et al., 2018), which can bias analyses of population structure (Goldberg & Waits, 2010), we genotyped a maximum of 2–3 individuals from small containers. In Madinah and Najran, there were fewer, but larger, containers so up to 5 individuals were genotyped from each container to obtain adequate population sample sizes. Relatedness among individuals from each location was tested using the maximum‐likelihood method in ML‐RELATE (Kalinowski et al., 2006).

A total of 389 individual mosquitoes were genotyped for 17 microsatellite markers according to established protocols (Brown et al., 2011; Slotman et al., 2007) (Additional file 1: Table S1). Although previous studies used 10–12 loci, increasing the number of loci (and particularly having more variable loci) is likely to increase the power of population genetic inferences compared to increasing the number of individuals (Landguth et al., 2012). The 17 loci were amplified using three multiplex PCR assays developed in this study (Set 1: B2, AG1, AC4, AG2, CT2 and A9; Set 2: AC5, B3, A1, AG5, AC2 and AC1; and Set 3: AC7, AG3, AG4, AG7 and AT1). Each reaction consisted of GoTaq G2 Colorless Master Mix 12.5 μL and 2.5 μL of 10X primer mix (2 μM each), 1 μL of DNA template (diluted 1:2) and 9 μL of sterile water to a total volume of 25 μL. PCR cycling conditions were as follows: an initial denaturation step at 95°C for 5 min, followed by 32 cycles of 95°C for 30 s, 60°C for 90 s and 72°C for 30 s with a 30 min final extension step at 60°C. For a few samples that did not show a complete profile, a repeat single‐plex reaction was performed. The genotyping was performed at Eurofins Genomics, Germany, on an ABI 3130xl (Eurofins Genomics). All PCR products were diluted with RNase‐free water at 1:300 dilution, and 1 μL of the diluted PCR product was mixed with 10 μL Hi‐Di formamide and 0.15 μL of LIZ‐500 internal size standard for loading onto the machine. The results were scored using GeneMapper 5.0 software (Applied Biosystems).

### 2.4 Genetic analysis and population structure

Micro‐Checker v2.2.3 (Van Oosterhout et al., 2004) was used to estimate the prevalence of microsatellite null alleles. The linkage disequilibrium (LD) tests were estimated among all possible pairs of the 17 loci in each population for a total of 13 populations using GENEPOP v4.2 (Rousset, 2008) (dememorization: 10,000; batches: 100; and iterations per batch: 10,000). In Excel calculator v1.2 (Gaetano, 2013), LD significance levels for multiple testing were corrected using the Holm‐Bonferroni sequential correction to adjust the *p*‐value to minimize type I error.

Summary statistics for microsatellite data were estimated using GenAlEx v6.5 (Peakall & Smouse, 2006). Observed heterozygosity (*H*_O_), expected heterozygosity (*H*_E_), population pairwise *F*_ST_ analysis, isolation by distance (Mantel test), the analysis of molecular variance (AMOVA), allelic richness (*AR*) and inbreeding coefficients (*F*_IS_) were calculated using Arlequin v3.5.2.2 (Excoffier & Lischer, 2010).

Two separate analyses for STRUCTURE were carried out: the first comprised 11 populations from only Saudi Arabia, and the second contained 13 populations from all of Saudi Arabia, Uganda and Thailand (Table 1). The genetic clusters and potential ancestry were identified using the Bayesian assignment algorithm implemented in STRUCTURE software v.2.3 (Pritchard et al., 2000). Twenty independent iterations were run with assumed populations ranging from *K* = 1 to *K* = 11 in the Saudi Arabia analysis and *K* = 1 to *K* = 13 when Uganda and Thailand were added. The calculation model for both the 11 and 13 population analyses was set as admixture ancestry with 100,000 burn‐in steps with 1,000,000 MCMC replicates. Following this, the optimum *K* value was estimated using Evanno's delta K model (Evanno et al., 2005) in STRUCTURE Harvester (https://taylor0.biology.ucla.edu/struct_harvest/). For better visualization of clustering plots, clumpak (http://clumpak.tau.ac.il/index.html) was used with the LargeKGreedy algorithm search method.

For complementary estimations of clustering, principal component analysis (PCA) and discriminant analysis of principal components (DAPC) were conducted in R using the *Adegenet* package (Jombart, 2008). DAPC grouping is obtained by maximizing the differences between the given populations.

To characterize gene flow (Nm, the number of migrants per generation) between groups, divMigrate‐online was used to draw a network depicting estimated gene flow values among populations with the setting of *α* = 0.05; however, due to the small sample size in some populations, bootstraps were set to zero (https://popgen.shinyapps.io/divMigrate‐online/) (Sundqvist et al., 2016). This analysis was conducted for Saudi populations alone and Saudi populations together with Uganda and Thailand.

### 2.5 Admixture, history and demographic analyses

To test our hypothesis on colonization events and whether the genetic composition of *A. aegypti* in Saudi Arabia derives from introductions from both African and non‐African populations, we used approximate Bayesian computation (ABC) methods in DIYABC 2.1.0 (Cornuet et al., 2014). Population samples from Thailand and Uganda were used to represent global non‐African and African groupings, respectively, in these tests of genetic admixture. The DIYABC program tested multiple scenarios and the best‐supported scenario was chosen based on the highest posterior probability (*p*) (Cornuet et al., 2014). We tested three evolutionary scenarios. In all of these, the deepest split is between African and non‐African global populations, based on previous reports of genetic structure of global populations (Gloria‐Soria et al., 2016). The three scenarios tested were as follows: (1) African populations gave rise to Saudi Arabia populations; (2) populations from global domestic populations outside Africa gave rise to Saudi Arabia populations; and (3) both African and out‐of‐African populations gave rise to Saudi Arabia populations (admixture scenario). Each evolutionary scenario was tested three times using a different composition for the Saudi Arabia populations (based on the STRUCTURE plot), that is, (A) all 11 populations in Saudi Arabia, (B) Jazan highlands, and (C) Jeddah and Makkah. The (B) and (C) were selected to represent the divergent groupings within Saudi Arabia detected by the STRUCTURE analysis.

## 3 RESULTS

### 3.1 Marker analysis and genetic diversity

The relatedness analysis (maximum‐likelihood method in ML‐RELATE) estimated the percentage of first‐degree relatives (sib‐sib or parent‐offspring) to be very low in most population samples, from 0.7% in Jazan to 2.1% in Jeddah. The percentage of first‐degree relatives was higher in Najran and Madinah (5.8% and 6.7%, respectively) (Additional file 1: Table S2). This is likely due to sampling more larvae per container but also possible accurately reflects the smaller sampling area of these populations. Since even these values of relatedness are low, they are expected to minimally affect estimates of genetic population structure and gene flow.

A total of 337 *A. aegypti* from 11 populations in Saudi Arabia and 52 *A*. *aegypti* from populations in Uganda and Thailand were characterized (Table 1). The results revealed a total of 226 alleles across the 17 genetic loci in the Saudi Arabia populations. The number of alleles was highest (174) in the Jazan highland region and lowest in Najran (80). The microsatellite marker AG2 showed the highest number of alleles (36), while the AG1 marker revealed the lowest (6) (Additional file 1: Table S3). An average of 13.29 alleles per locus was observed in the Saudi Arabia populations. All 17 genetic loci were polymorphic in all populations, except for AC2 and CT2 which were monomorphic in one population (SahilE, *n* = 5).

There was a low percentage of null alleles in the data (0.00 to 0.20) across all loci (Additional file 1: Table S4). Since the presence of null alleles at frequencies lower than 0.20 does not influence estimates of genetic differentiation (Chapuis & Estoup, 2007; Gloria‐Soria, 2022; Wei et al., 2019), all loci were included in subsequent analysis.

Only one pair of loci, AG1 and AG3, showed significant linkage disequilibrium across all the Saudi Arabian and the Thailand populations but not the Ugandan samples after Holm‐Bonferroni sequential correction (Additional file 2). Brown et al. (2011) also found linkage disequilibrium for the two loci (AG1 and AG3) so removed one locus but this made no impact on the overall results (Brown et al., 2011). Both loci were retained by Rasheed et al., 2013 who detected no linkage disequilibrium and no physical linkage was reported between them when the markers were originally isolated (Slotman et al., 2007). We observed no substantial differences in our analyses when using both loci or when retaining only one, so we present analyses retaining both loci.

A summary of the genetic diversity over loci for each population is shown in Table 2. The average allelic richness (AR) was highest (4.50) in the Jazan highlands and lowest in Najran (3.19). For Uganda and Thailand, we observed an average of 7.40 and 4.82 alleles per locus, and the average allelic richness was 4.40 and 3.37, respectively. These values were higher in Uganda but lower in Thailand than in Saudi Arabia (3.37).

**TABLE 2:** Summary statistics of genetic diversity showing the mean and SE over all 17 microsatellite loci for each population.

Population	*N*	Na	*N*_E_	*I*	*H*_O_	*H*_E_	*uH*_E_	AR
Madinah								
Mean	42	5.176	2.782	1.128	0.531	0.574	0.581	3.32
SE		0.456	0.281	0.099	0.043	0.044	0.045	
Jeddah								
Mean	39	6.059	3.243	1.255	0.606	0.618	0.626	3.64
SE		0.976	0.433	0.125	0.042	0.039	0.039	
Makkah								
Mean	30	6.118	2.978	1.247	0.588	0.616	0.626	3.59
SE		0.747	0.261	0.101	0.038	0.036	0.036	
SahilW								
Mean	8	3.765	2.384	0.955	0.485	0.510	0.544	3.22
SE		0.379	0.244	0.100	0.055	0.046	0.049	
SahilM								
Mean	25	6.529	3.532	1.391	0.598	0.665	0.678	4.02
SE		0.703	0.374	0.105	0.037	0.035	0.035	
SahilE								
Mean	5	3.471	2.642	0.988	0.518	0.536	0.596	3.47
SE		0.333	0.264	0.118	0.079	0.058	0.065	
SahilAS								
Mean	22	6.647	3.418	1.383	0.589	0.650	0.666	4.07
SE		0.528	0.370	0.096	0.044	0.035	0.036	
Jazan villages								
Mean	72	8.706	3.447	1.408	0.540	0.649	0.654	3.93
SE		1.006	0.344	0.110	0.043	0.041	0.041	
Jazan lowland								
Mean	16	5.706	3.655	1.322	0.519	0.640	0.661	4.13
SE		0.706	0.492	0.132	0.057	0.047	0.048	
Jazan highland								
Mean	46	10.235	4.793	1.686	0.634	0.718	0.726	4.50
SE		1.305	0.685	0.129	0.041	0.041	0.041	
Najran								
Mean	32	4.647	2.686	1.060	0.475	0.552	0.561	3.19
SE		0.383	0.306	0.102	0.058	0.049	0.050	
Thailand								
Mean	22	4.824	2.767	1.127	0.561	0.593	0.610	3.37
SE		0.487	0.256	0.086	0.057	0.034	0.035	
Uganda								
Mean	30	7.706	4.267	1.543	0.588	0.704	0.717	4.40
SE		0.869	0.591	0.113	0.048	0.035	0.036	

Abbreviations: AR, allelic richness; *H*_E_, expected heterozygosity; *H*_O_, observed heterozygosity; *I*, Shannon's information index; *N*, sample size for each population; Na, average number of alleles per locus; *N*_E_, effective population size; SE, standard error; *uH*_E_, unbiased expected heterozygosity.

### 3.2 Isolation by distance (IBD) within Saudi Arabia

Results of the Mantel test revealed no significant correlation between genetic and geographical distance (*r* ^2^ = 0.007, *p* = 0.53) (*n* = 337) in Saudi Arabia (Additional file 1: Figure S2). No correlation was observed when the Jazan highlands or Najran populations were excluded. When Mantel tests were performed at the local scale, within each population, a significant positive but low correlation (*r* ^2^ = 0.225, *p* < 0.0001) was observed in the Najran population (*n* = 32) but not in any other population (Additional file 1: Figure S2).

### 3.3 Population structure and clustering analysis in Saudi Arabia

**FIGURE 3:**
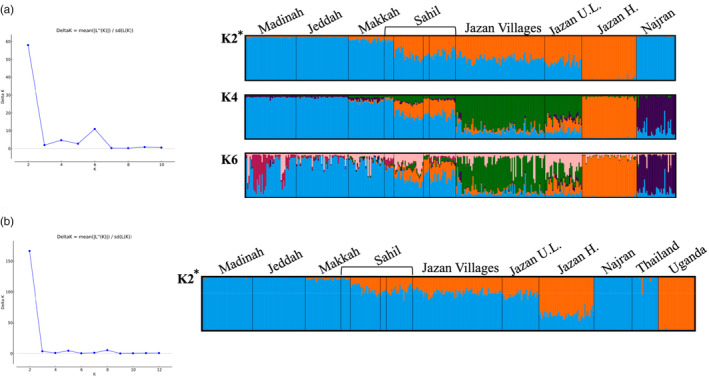
Population structure analysis of *Aedes aegypti* based on 17 microsatellite loci using STRUCTURE from (a) eleven populations from Saudi Arabia (*K* = 2) and (b) eleven populations from Saudi Arabia, one population from Thailand and one population from Uganda (*K* = 2). Each colour indicates a distinct genetic grouping, and each vertical line represents an individual with the proportion of population ancestry from each genetic grouping in that individual indicated by the height of the colour. To the left of the STRUCTURE plots is the graph showing estimation of the optimal *K* value according to the Evanno et al. (2005) method, with the optimal estimated value indicated by * next to the structure plot.

The analysis of molecular variance (AMOVA) results showed a very low genetic population structure with a percentage of variation among populations of only 4.87% (Table 3). This increased to 11.57% among individuals, and the highest percentage of genetic variation was observed at the individual level (83.56%) (Table 3). The pairwise genetic differentiation (*F*_ST_) matrix of the 11 populations of *A. aegypti* in Saudi Arabia is shown in Table 4. Pairwise *F*_ST_ estimates were low, ranging between 0.014 and 0.161, but most of the *F*_ST_ values were significant. Bayesian clustering analysis using STRUCTURE (Pritchard et al., 2000) identified *K* = 2 as the optimal number of genetic clusters for the 11 populations in Saudi Arabia (Figure 3a). This was supported by an extremely high delta K value which changed almost to zero at values of *K* = 3 onwards, indicating two strongly differentiated genetic groupings with little genetic structuring beyond this. The Jazan highland population was genetically distinct as one group (orange colour, Figure 3a), while the western regions (Makkah, Jeddah and Madinah) and Najran comprised the second group (blue colour, Figure 3a). The Sahil and lowland of Jazan show admixture of genetically distinct clusters (Figure 3a). The optimal number of clusters identified by STRUCTURE remained at two, again with extremely strong support from the delta K values, even when the Thailand and Ugandan populations were included in the analysis (Figure 3b). As Thailand and Uganda were completely distinct from each other and each shared cluster membership with Saudi Arabian populations, it indicates that populations from Saudi Arabia have genetic ancestry from both the ancestral African and the pantropical domestic forms. Individuals from western Saudi Arabia and Najran clustered with Thailand (representing the domestic form), whereas individuals from Sahil and Jazan showed varying levels of admixture with individuals having genetic similarity to both pantropical and African groupings. The Jazan highlands had the greatest proportion of ancestry corresponding to Africa. At *K* = 3, *A. aegypti* from Najran became genetically distinct. Higher *K* values detected additional subtle genetic structuring within Saudi Arabia, including the admixed Jazan lowland and village populations at *K* = 4 and Madinah as a separate grouping at *K* = 6. The STRUCTURE plots for *K* = 3 to 6 are presented in Additional file 1: Figure S3.

**TABLE 3:** AMOVA analysis of the genetic variation within and among *Aedes aegypti* populations from Saudi Arabia.

Source of variation	df	Sum of squares	Estimated variance	% of variation	*p*‐value
Among populations	10	235.65	0.276	4.87	0.000
Among individuals	349	2112.42	0.656	11.57	0.000
Within individuals	360	1706.50	4.740	83.56	0.000
Total	719	4054.57	5.673	100	

Abbreviation: df, degrees of freedom.

**TABLE 4:** Pairwise *F*_ST_ matrix estimates for *Aedes aegypti* populations in Saudi Arabia.

Population	Mad	Jed	Mak	SahW	SahM	SahE	SahA	JazV	JazL	JazH
Madinah										
Jeddah	0.028									
Makkah	0.030	0.016								
SahilW	**0.035**	**0.030**	0.053							
SahilM	0.042	0.032	0.031	0.035						
SahilE	0.101	0.068	0.078	0.106	0.084					
SahilAS	0.032	0.020	0.021	0.043	0.024	0.064				
Jazan villages	0.047	0.034	0.030	0.056	0.030	0.064	0.021			
Jazan lowland	0.054	0.034	0.035	0.060	**0.014**	0.089	0.026	0.023		
Jazan highland	0.107	0.091	0.085	0.109	0.049	0.077	0.056	0.050	0.039	
Najran	0.084	0.063	0.073	0.115	0.105	0.162	0.082	0.086	0.098	0.161

*Note*: *F*_ST_ estimates in bold have *p*‐values ranging from 0.009 to 0.035.

**FIGURE 4:**
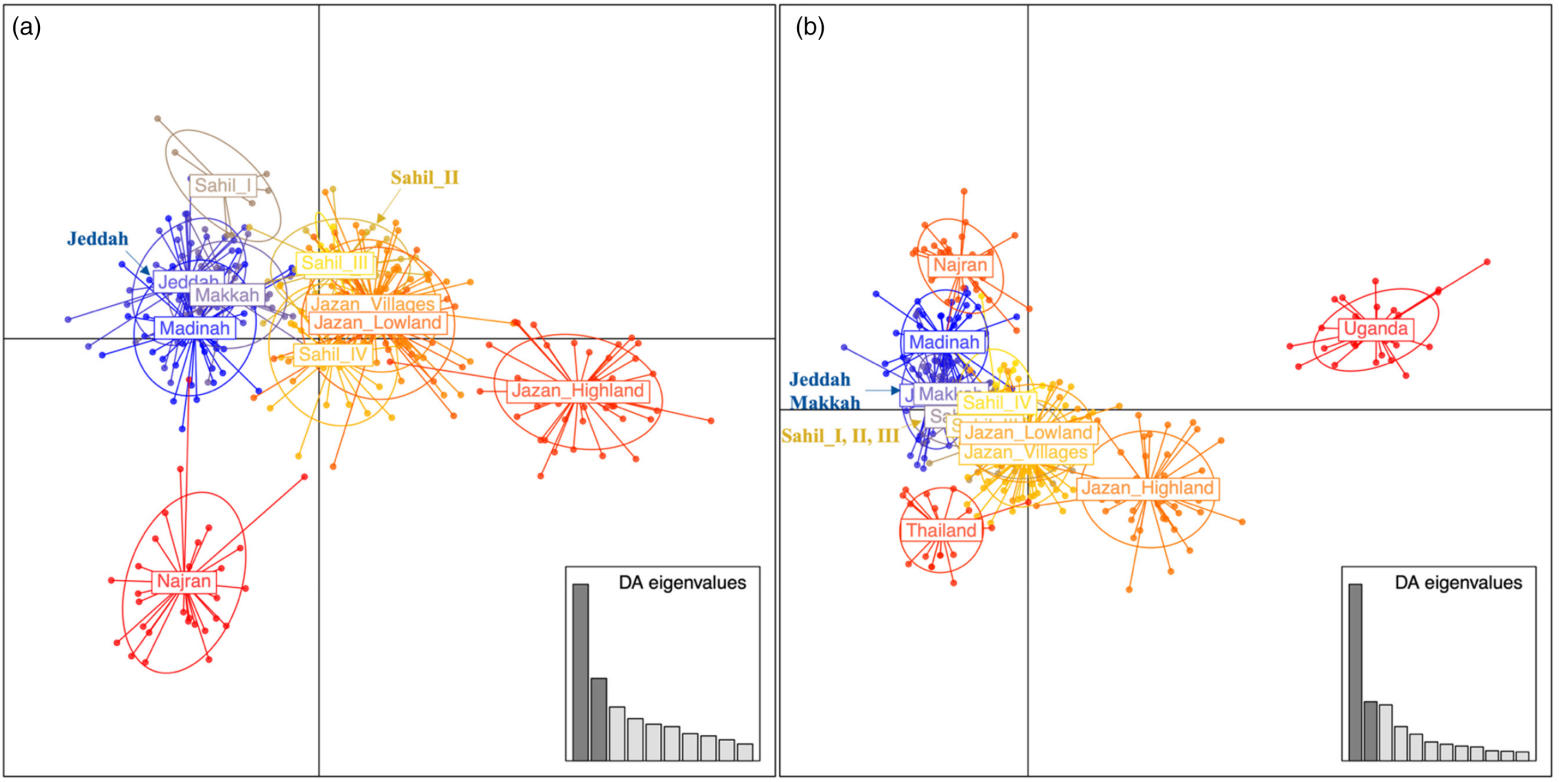
The discriminant analysis of principal component (DAPC) clustering analysis based on 17 microsatellite loci for *Aedes aegypti* populations from (a) Saudi Arabia (11 populations) and (b) Saudi Arabia, Thailand and Uganda (13 populations) using *Adegenet* (Jombart, 2008). Each clustering colour corresponds to a population, and dots represent individuals. A bar plot of the discriminant analysis eigenvalues corresponds to the variance ratio between groups. Sahil_I = SahilW; Sahil_II = SahilM; Sahil_III = SahilE; Sahil_IV = SahilA.

Discriminant analysis of principal components (DAPC) and principal component analysis (PCA) on Saudi Arabian populations grouped them into four clusters corresponding broadly to geography (Figure 4a and Additional file 1: Figure S4a). The Jazan highlands and Najran each formed a distinct cluster, the western populations (Madinah, Makkah, Jeddah) clustered together, and the remaining Jazan and Sahil populations (except Sahil_I (SahilW)) clustered together. To further explore the relationship of Saudi Arabia populations to African and Southeast Asian populations, a DAPC and PCA including Thailand and Uganda were performed (Figure 4b and Additional file 1: Figure S4b). All the Saudi Arabia populations were positioned more closely to Thailand than Uganda but the Jazan highlands was positioned between Thailand and Uganda. The PCA results were consistent with the DAPC clustering (Additional file 1: Figure S4a,b).

### 3.4 Gene flow (*Nm*) network

**FIGURE 5:**
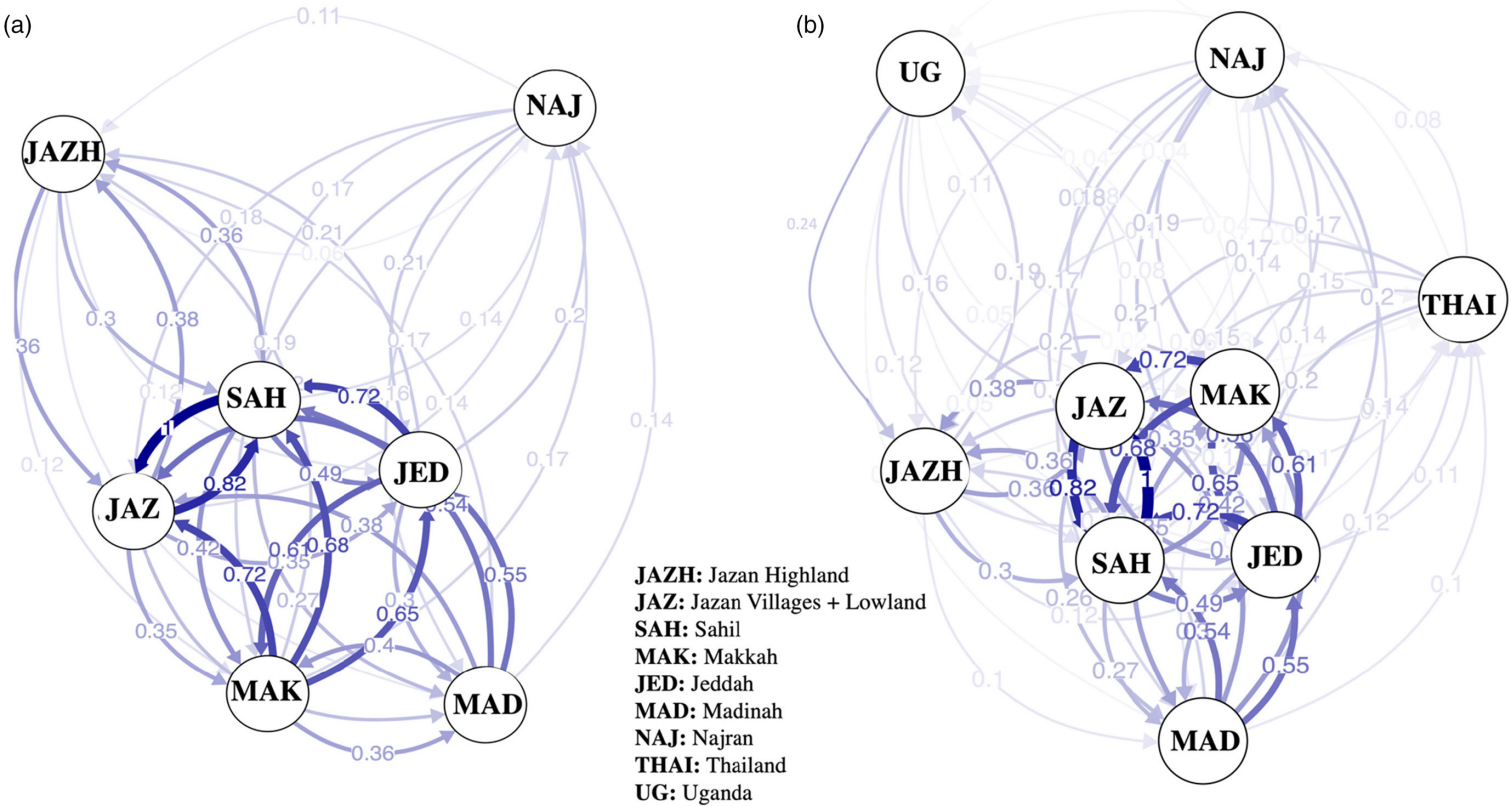
Network of directed migration routes using divMigrate with *α* = 0.05. Each circle with a code represents a population which is connected with arrows with the numbers representing the migration values, *Nm* (number of migrants per generation). The weight of the line corresponds to the extent of migration. (a) The gene flow of seven Saudi Arabia study populations; (b) The network of seven Saudi Arabia, one Uganda and one Thailand populations.

A high migration rate was detected within the western regions (between Makkah, Jeddah and Madinah) as well as between the Jazan lowland and Sahil regions (Figure 5a). Jazan highland and Najran were both genetically isolated from other populations. When Uganda and Thailand were included, the network revealed some connectivity of Uganda with the Jazan highland and some connectivity of Thailand with the western region, albeit low in both cases. The strength of gene flow within the western region, and between Sahil and Jazan, remained high (Figure 5b).

### 3.5 Demographic analysis and population history

**FIGURE 6:**
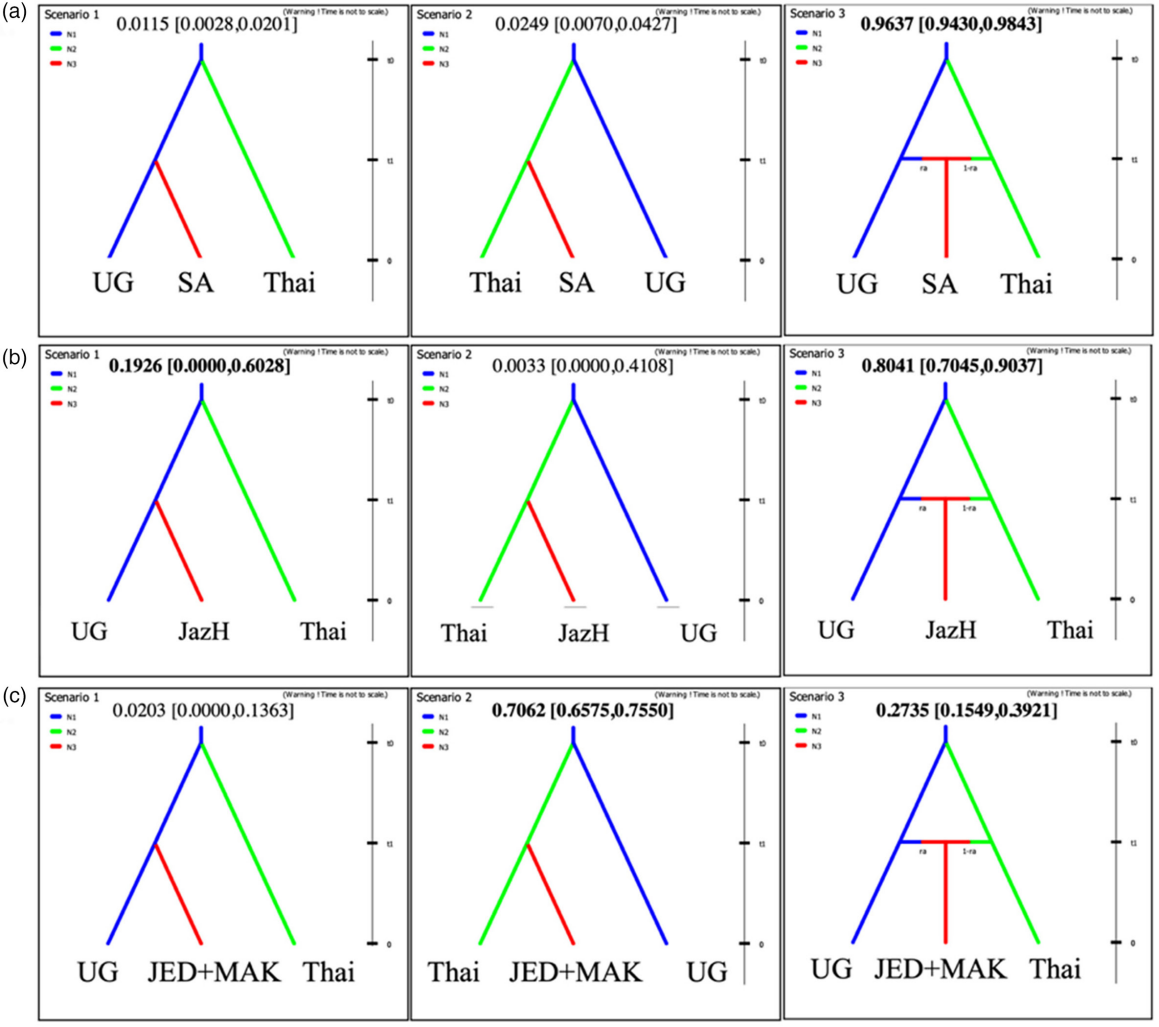
Three evolutionary scenarios used to test alternative hypotheses of colonization and admixture of *Aedes aegypti* in Saudi Arabia using DIYABC software of Cornuet et al. (2014) using microsatellite data from Saudi Arabia (SA) populations, the Thailand (Thai) population to represent the global domestic form and Uganda (UG) to represent the ancestral African form. From left to right in each panel, the evolutionary scenarios are as follows: colonization of SA by the ancestral African form only; colonization of SA by the global domestic form only; and colonization of SA by both forms with admixture. This set of three evolutionary scenarios was tested using different populations from Saudi Arabia: (a) all populations from Saudi Arabia (SA); (b) Jazan highlands (JazH) only; and (c) Jeddah and Makkah only (JED+MAK).

Genetic admixture was observed in Sahil and some of the Jazan collections (Figure 3a); therefore, evolutionary scenarios were tested three times using different populations for the Saudi Arabia regions. First, when the evolutionary scenarios considered all populations of Saudi Arabia as representatives of Saudi Arabian *A. aegypti*, there was extremely strong support for genetic admixture involving African and non‐African populations (posterior probability, *p* = 0.9637) (Figure 6a). Second, when the analysis was run using only the Jazan highland population (Figure 6b), the admixture scenario was still supported but the posterior support probability was lower (*p* = 0.8041, Figure 6b). This was due to significant support being given to the model of Jazan having descended from African populations only (*p* = 0.1926, Figure 6b). Third, when the evolutionary scenarios included only the western (Jeddah and Makkah) populations, the best‐supported scenario was for a sole origin of the Saudi Arabian populations from the global domestic form represented here by Thailand (*p* = 0.7062) (Figure 6c).

Overall, these data provide strong support for Saudi Arabia populations of *A. aegypti* being the result of genetic admixture, but they also indicate that admixture proportions vary geographically across Saudi Arabia with the Jazan highland having a greater proportion of genetic ancestry from Africa and the western regions having a great proportion from non‐African populations. Details on the posterior probabilities and split time between regions used as priors for the ABC analysis and inferred for the supported scenarios are provided in Additional file 1: Table S5.

## 4 DISCUSSION

The present study significantly expands upon previous studies of *A. aegypti* genetic population structure in Saudi Arabia that used mitochondrial and ribosomal DNA markers (Khater et al., 2021) by using 17 microsatellite markers and by covering a wider geographic region. This study covers all six geographic regions where dengue has been reported in Saudi Arabia: Jazan, Sahil, Jeddah, Makkah, Madinah and Najran (Ministry of Health, 2016). Further, using comparisons with an African and a Southeast Asian population to represent the ancestral form of *A. aegypti* from Africa and the pantropical domestic form, respectively, enabled us to elucidate the genetic ancestry of *A. aegypti* in Saudi Arabia.

The overall level of genetic differentiation among the studied regions within Saudi Arabia was low. This, together with the absence of a correlation between genetic and geographic distance, is consistent with long‐distance passive dispersal of *A. aegypti* in Saudi Arabia. Passive dispersal through human transportation (largely in the form of immature stages or eggs) is commonly inferred in *A. aegypti* (Carvajal et al., 2020; Fernando et al., 2020; Hlaing et al., 2010; Maffey et al., 2020, 2022; Rasheed et al., 2013). This has generally been attributed to facilitation by human movement, particularly by road. The volume of automobiles moving through the Sahil region (from Jazan to Makkah and Jeddah) is high which corresponds to the particularly high levels of migration of *A. aegypti* inferred to occur between these regions. Conversely, the low road connectivity towards Najran from the other locations studied here may be responsible for the slightly higher genetic isolation of Najran. Overall, the patterns of genetic differentiation and migration detected here and their correlation with road connectivity are consistent with transportation networks facilitating the passive dispersal of *A. aegypti* in Saudi Arabia (Figure 1). This could facilitate the spread of pathogens and insecticide resistance to new locations in the country, that is, north or east.

The study of the population structure of *A. aegypti* in Saudi Arabia revealed that there are two main genetic groupings within Saudi Arabia with extensive genetic admixture in some locations. The similarity of these genetic groupings to ancestral African populations (represented by Uganda) or the global domestic form (represented by the Thailand form) together with the ABC analysis indicates that Saudi Arabia has been colonized by *A. Aegypti* from Africa as well as by the pantropical domestic form. Clustering of the western populations with Thailand in the present study is consistent with a previous global diversity study (Gloria‐Soria et al., 2016), where Jeddah was clustered with Pakistan or the New World. These populations in Saudi Arabia also had low genetic diversity similar to that observed in the Thailand population. Together, this indicates that *A. aegypti* in this part of Saudi Arabia is indistinguishable from global domestic populations of *A. aegypti*. This might be explained by the large amount of international shipping coming into Jeddah as well as the popularity of Makkah. Every year, more than two and a half million people gather in Makkah (Mecca) from over 180 nations across the world (particularly Southeast Asia) to practise their religion (Hajj and Umrah), arriving through the international airport in Jeddah (Haridi et al., 2019; Zayed et al., 2017).

In contrast, the STRUCTURE analyses revealed that the Jazan highlands was genetically distinct from other populations in Saudi Arabia having more genetic similarity with Uganda than western populations of Saudi Arabia. The highest genetic diversity of the Saudi Arabian populations was in the Jazan highlands which had genetic diversity as high as that of Uganda. This information is consistent with the ABC analysis which indicates that, although the Jazan highlands population still has a signal of genetic admixture, that much of the ancestry in this region stems from dispersal directly from sub‐Saharan Africa. This is further supported by the observation made during collections in the Jazan highlands that mosquitoes from this area were dark in colour as is *A. aegypti formosus* from African forests. The STRUCTURE and ABC analyses indicated genetic admixture between the two genetic groupings originating from Africa or the global population in the intervening geographic regions of Sahil and the Jazan region. The mixing of these two distinct genetic forms of *A. aegypti* has also recently been inferred to have occurred in Sudan (Elnour et al., 2022).

The retention of the high genetic diversity of *A. aegypti* in the Jazan highlands indicates that it is the result of historical migration involving a larger number of founders, potentially repeated migration over a long time period, since there is no evidence of a genetic bottleneck. The African‐like population in the Jazan highlands may therefore have been introduced through Yemen as Jazan shares a border with Yemen and is close to some parts of Eastern Africa. Although *A. aegypti* was first recorded in the Arabian Peninsula area (covering Saudi Arabia and Yemen) in the mid‐20th century (Mattingly & Knight, 1956), Yemen is known to have the oldest port in the region (Aden), which traded with Eastern Africa and Southern Asia about 2000–3000 years ago (Martin & Vigne, 1997). Further investigations using Yemeni samples would allow this hypothesis of entry through Aden to be tested. Nonetheless, current shipments from Africa largely use the port of Jeddah (Waters, 2017); therefore, further analyses are needed to determine whether *A. aegypti* is still entering the Arabian Peninsula and if so via which route(s)? This knowledge is important as it could inform surveillance efforts to detect pathogens that might be imported from Africa. The biological characteristics of the genetically distinct population in the Jazan highlands also require further investigation. In particular, the presence of a unique African‐like population in the Jazan highlands might differ in disease transmission ability compared to non‐African populations (Dickson et al., 2014; Weetman et al., 2018). Therefore, findings of the present study should be taken into account when designing vector control strategies, like potential future trials of *Wolbachia* releases for dengue control.

## FUNDING INFORMATION

This research was funded by Jazan University, Saudi Arabia, and Saudi Arabia Cultural Bureau in London and Research England QR GCRF allocation to the University of Manchester.

## CONFLICT OF INTEREST STATEMENT

The authors declare that they have no competing interests.

## Supporting information


Additional file 1.

Additional file 2.

Additional file 3.

## Data Availability

The raw microsatellite data for this study are available at: https://vectorbase.org—bioproject ID VBP0000839 (Giraldo‐Calderón et al., 2015) and are provided in Additional file 3.
