# Correction to Microsatellite‐based analysis reveals *Aedes aegypti* populations in the Kingdom of Saudi Arabia result from colonization by both the ancestral African and the global domestic forms

**DOI:** 10.1111/eva.13745

**Published:** 2024-07-01

Mashlawi, A. M., Alqahtani, H., Abuelmaali, S. A., Gloria‐Soria, A., Saingamsook, J., Kaddumukasa, M., … Walton, C. (2024). Microsatellite‐based analysis reveals *Aedes aegypti* populations in the Kingdom of Saudi Arabia result from colonization by both the ancestral African and the global domestic forms. *Evolutionary Applications*, *17*(2), e13661.

The authors would like to amend the Acknowledgments section of the above article and consolidate some of the resources mentioned in it. The Acknowledgments should read as follows:

