# Supplementary material for: Microsatellite‐based analysis reveals Aedes aegypti populations in the Kingdom of Saudi Arabia result from colonization by both the ancestral African and the global domestic forms
Source: Evol Appl. 2024 Feb 22;17(2):e13661. doi: 10.1111/eva.13661 (PMC10883788; doi:10.1111/eva.13661)
Supplement: Supplementary file 1 — Additional file 1. [file EVA-17-e13661-s003.docx]

Additional file 1 (Appendix)


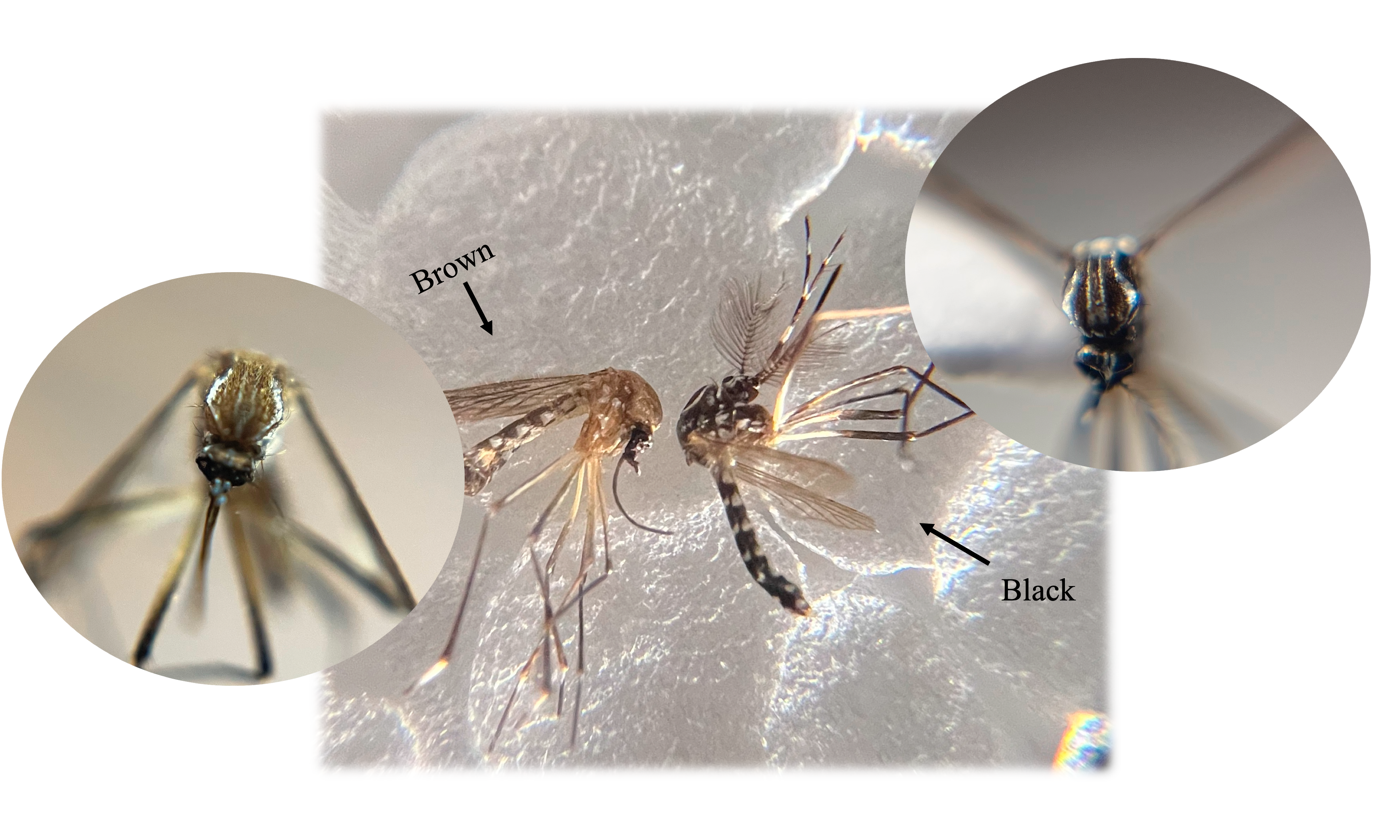


**Figure S1.** Lateral and thorax dorsal photomicrographs view show the two forms (pale and dark) observed in Saudi Arabia.

**Table S1.** The 17 microsatellite loci used in the population structure study. The size range is derived from Slotman et al. (2007) and Brown et al. (2011).

| Locus | Motif | Primer 5'-3' | Fluorescent dye | Size Range |
| --- | --- | --- | --- | --- |
| AC2 | Di- | F-AATACAACGCGATCGACTCC  R-AACGATTAGCTGCTCCGAAA | FAM | 176-190 |
| AC5 | Di- | F-TGGATTGTTCTTAACAAACACGAT  R-CGATCTCACTACGGGTTTCG | FAM | 149-164 |
| AC7 | Di- | F-TCGGCAAATTACCACAAACA  R-CATTGGACTCGCTATAACACACA | FAM | 129-143 |
| AG3 | Di- | F-CGCCAAAACTGAAAACTGAA  R-AAGGGCGGTGATGACTTTCT | FAM | 164-178 |
| B2 | Tri- | F-GGAAACACTTGCAGGGACAT  R-GCAGATGGTGGCAGTAGTGA | FAM | 95-119 |
| AG1 | Di- | F-AATCCCCACACAAACACACC  R-GGCCGTGGTGTTACTCTCTC | YAKYE | 113-129 |
| AG4 | Di- | F-AAAACCTGCGCAACAATCAT  R-AAGGACTCCGTATAATCGCAAC | YAKYE | 147-169 |
| B3 | Tri- | F-GCAAGTTGCAAAGTGCTCAA  R-ACCCACCGTTTGCTTTGTAG | YAKYE | 148-181 |
| CT2 | Di- | F-CGCAGTAGGCGATATTCGTT  R-ACCACCACCAACACCATTCT | YAKYE | 184-192 |
| A9 | Tri- | F-GCAGCATGCACTTCACATTT  R-CGAATGGCATCTGATTCAAG | ATTO565 | 179-203 |
| AC4 | Di- | F-GCGAATCGGTTCCCATAGTA  R-CTTTATCGATCGACGCCATT | ATTO565 | 128-130 |
| AG5 | Di- | F- TGATCTTGAGAAGGCATCCA  R-CGTTATCCTTTCATCACTTGTTTG | ATTO565 | 170-180 |
| AT1 | Di- | F-CGTCGACGTTATCTCCTTGTT  R-GGACCGGAAAGACACAGACA | ATTO565 | 156-174 |
| A1 | Tri- | F-GACGTAAACCGAGTGGGAGA  R-GCATTTAACCGCGCTAGAAC | ATTO550 | 149-177 |
| AC1 | Di- | F-TCCGGTGGGTTAAGGATAGA  R-ACTTCACGCTCCAGCAATCT | ATTO550 | 193-209 |
| AG2 | Di- | F-TCCCCTTTCAAACCTAATGG  R-TTTGCCCTCGTATGCTCTCT | ATTO550 | 115-178 |
| AG7 | Di- | F-CGTGCGAGTGAATGAGAGAC  R-CATCCTCTCATCAGCTTCTAATAAA | ATTO550 | 153-185 |
|  |  |  |  |  |

**Table S2.** Summarizing the relatedness by Maximum Likelihood (R) using ML-Relate software across *Aedes aegypti* of all Saudi Arabia populations.

| **Population** | **No. indivi** | **Grand Total R** | **No. Of individual in each relationship in R** | | | | **%** |
| --- | --- | --- | --- | --- | --- | --- | --- |
|  |  |  | **FS** | **HS** | **PO** | **U** | **(FS+PO)** |
| **Jazan** | 147 | 10731 | 71 | 1021 | 9 | 9630 | **0.7** |
| Sahil | 45 | 990 | 12 | 70 | 1 | 907 | **1.3** |
| Makkah | 30 | 435 | 5 | 42 | 0 | 388 | **1.1** |
| Jeddah | 43 | 903 | 15 | 92 | 4 | 792 | **2.1** |
| Madinah | 42 | 861 | 45 | 69 | 13 | 734 | **6.7** |
| Najran | 32 | 496 | 22 | 33 | 7 | 434 | **5.8** |

_The FS = full sibling, HS = half-sib, PO = parent /offspring and U = unrelated; (FS+PO) % percentage of the first-degree relatives ( (FS + PO/Total * 100); Grand Total = overall number of relationships between each two individuals in R; R = The relationship with the highest likelihood_

**Table S3.** Number of alleles per locus per population in Saudi Arabia, Thailand and Uganda.

| Locus | Jazan  (Total) (n=164) |  | Jazan- Highland (n=46) | Jazan w/o Highland (n=118) | Sahil (n=60) | Makkah (n=30) | Jeddah (n=43) | Najran (n=32) | Madinah (n=42) | Thailand  (n=22) | Uganda  (n=30) | Total |
| --- | --- | --- | --- | --- | --- | --- | --- | --- | --- | --- | --- | --- |
| AC2 | 8 |  | 7 | 6 | 5 | 5 | 4 | 4 | 4 | 4 | 8 | **55** |
| AC5 | 19 |  | 18 | 15 | 11 | 8 | 10 | 6 | 8 | 6 | 15 | **116** |
| AC7 | 9 |  | 6 | 8 | 9 | 7 | 7 | 5 | 5 | 4 | 9 | **69** |
| AG3 | 9 |  | 7 | 8 | 8 | 5 | 6 | 5 | 5 | 4 | 7 | **64** |
| B2 | 5 |  | 5 | 5 | 6 | 4 | 4 | 3 | 4 | 3 | 8 | **47** |
| AG1 | 6 |  | 5 | 6 | 5 | 4 | 4 | 4 | 4 | 5 | 6 | **49** |
| AG4 | 12 |  | 11 | 9 | 6 | 5 | 4 | 3 | 4 | 5 | 10 | **69** |
| B3 | 12 |  | 11 | 8 | 7 | 4 | 4 | 4 | 3 | 5 | 6 | **64** |
| CT2 | 9 |  | 7 | 8 | 7 | 3 | 3 | 4 | 4 | 3 | 6 | **54** |
| A9 | 8 |  | 8 | 7 | 6 | 5 | 4 | 2 | 4 | 5 | 7 | **56** |
| AC4 | 12 |  | 8 | 9 | 4 | 2 | 2 | 3 | 3 | 2 | 5 | **50** |
| AG5 | 10 |  | 10 | 8 | 7 | 8 | 7 | 6 | 7 | 3 | 7 | **73** |
| AT1 | 12 |  | 10 | 11 | 10 | 11 | 8 | 7 | 7 | 9 | 8 | **93** |
| A1 | 8 |  | 8 | 7 | 8 | 6 | 5 | 4 | 5 | 3 | 9 | **63** |
| AC1 | 11 |  | 11 | 9 | 9 | 4 | 4 | 5 | 4 | 5 | 10 | **72** |
| AG2 | 29 |  | 16 | 26 | 23 | 14 | 19 | 9 | 9 | 7 | 7 | **159** |
| AG7 | 30 |  | 26 | 20 | 14 | 9 | 10 | 6 | 8 | 9 | 21 | **153** |
| Total | **209** |  | **174** | **170** | **145** | **104** | **105** | **80** | **88** | **82** | **149** | **1306** |

**Table S4.** Null allele frequency at all the 17 genetic loci calculated by MICRO-CHECKER software with different methods. ≥ 0.20 frequency of null allele is shown in bold.

| Locus | Oosterhout | Brookfield 1 | Brookfield 2 |
| --- | --- | --- | --- |
| AC2 | 0.0232 | 0.0119 | 0.0752 |
| AC5 | 0.0497 | 0.042 | 0.0683 |
| AC7 | 0.0274 | 0.0164 | 0.0549 |
| AG3 | 0.0441 | 0.0366 | 0.0867 |
| B2 | 0.0885 | 0.0546 | 0.0839 |
| AG1 | **0.2041** | 0.1829 | **0.2067** |
| AG4 | 0.0787 | 0.0662 | 0.0853 |
| B3 | 0.0227 | 0.0167 | 0.0574 |
| CT2 | -0.0066 | -0.0025 | 0.0399 |
| A9 | 0.1294 | 0.0933 | 0.1716 |
| AC4 | 0.1134 | 0.0878 | 0.1116 |
| AG5 | 0.1127 | 0.1037 | 0.1544 |
| AT1 | 0.055 | 0.0491 | 0.0876 |
| A1 | 0.1357 | 0.1198 | 0.1479 |
| AC1 | 0.0306 | 0.0235 | 0.0512 |
| AG2 | 0.0777 | 0.0716 | 0.0868 |
| AG7 | 0.0757 | 0.0671 | 0.0908 |


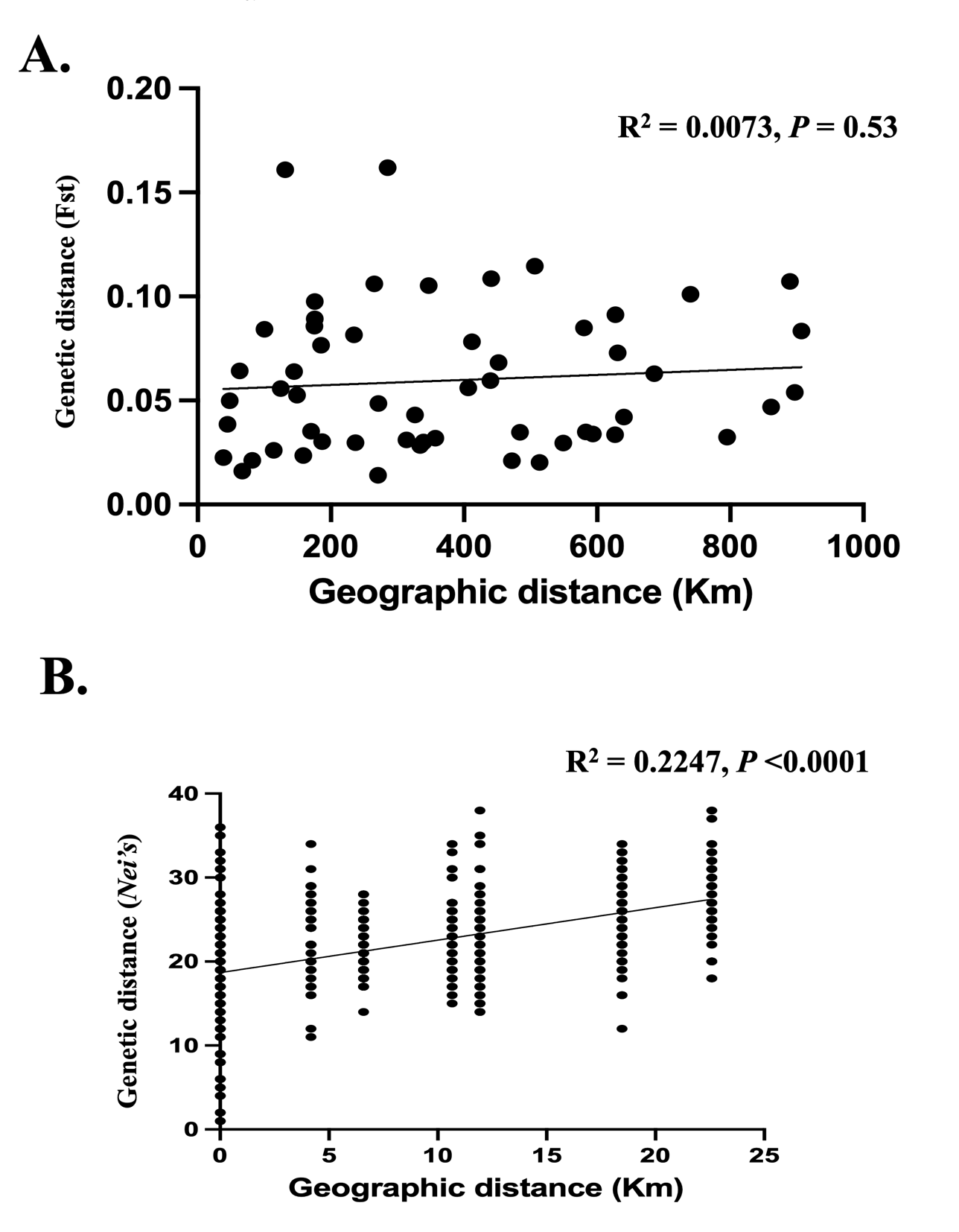


**Figure S2.** Correlation between genetic and geographic distances among **A)** all the 11 populations from Saudi Arabia (population-level) and **B)** the Najran population (individual-level).

**
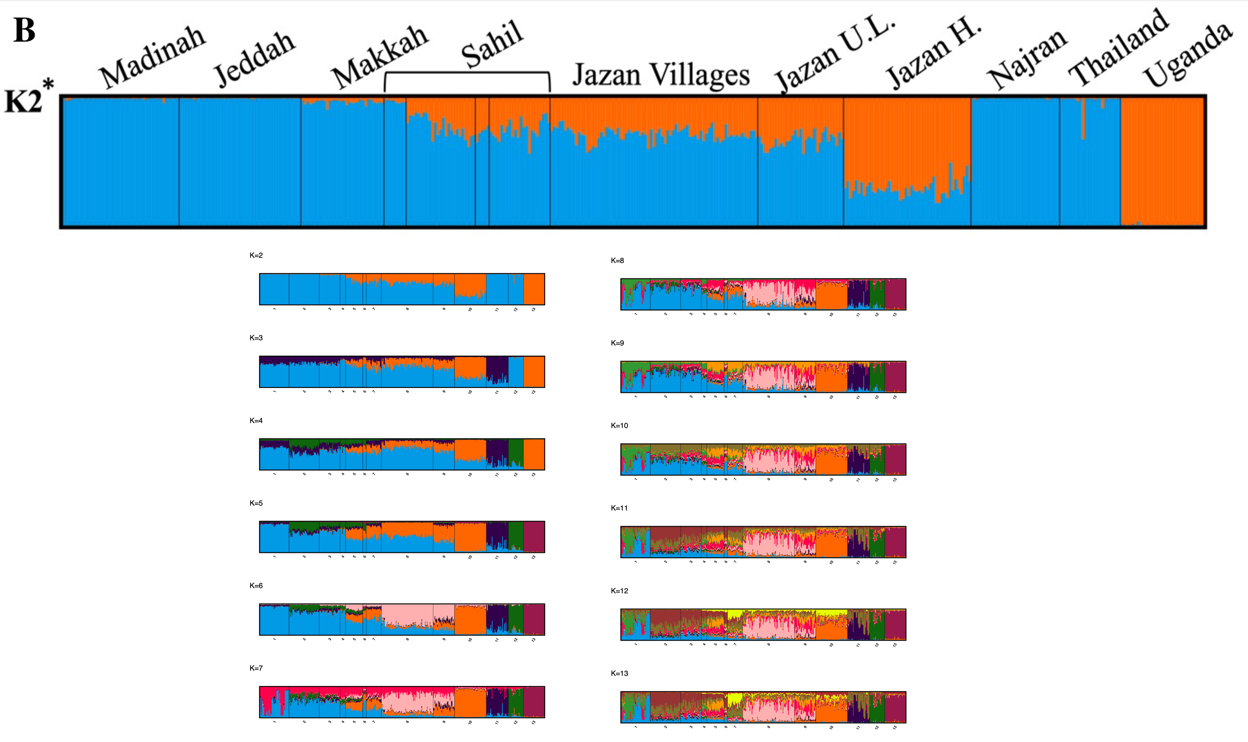
**

**
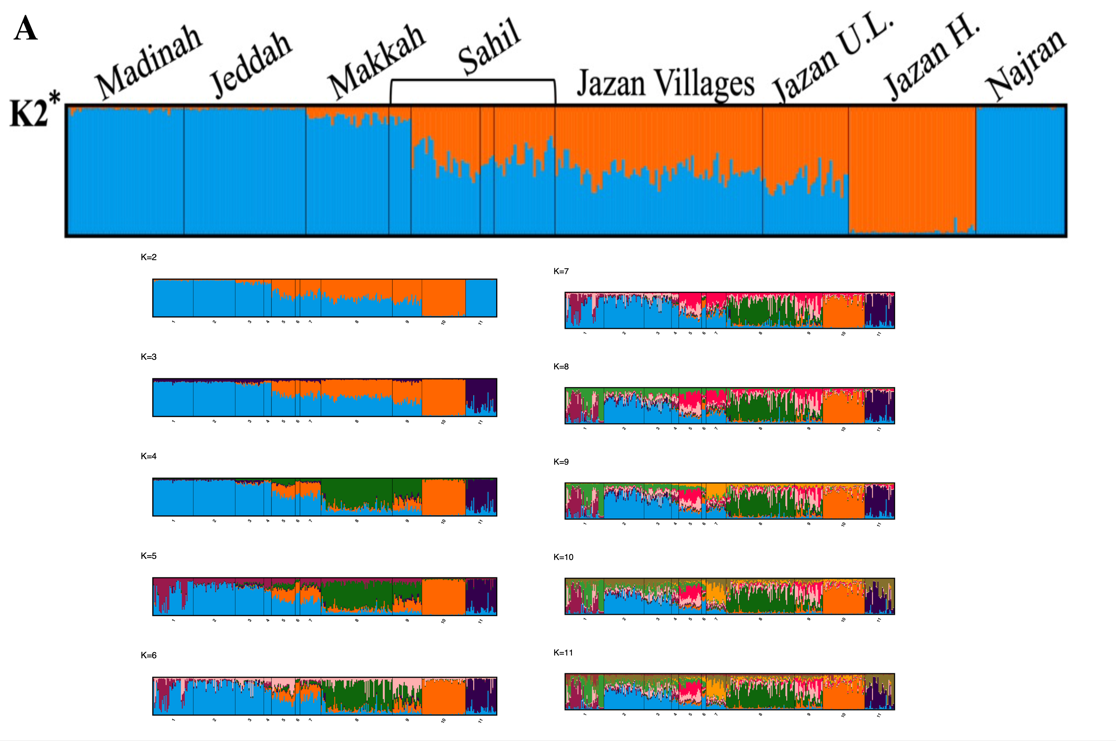
**

**Figure S3.** STRUCTURE. Population structure analysis of *Ae. aegypti* from eleven populations; Saudi Arabia ((A) K = 2-11 and (B) K = 2-13) based on 17 microsatellite loci using STRUCTURE software (Pritchard et al., 2000). Each cluster/colour indicates populations/ subpopulations, and each bar represents an individual. The height of the color bar indicates the percentage of the ancestry of each cluster for a particular individual. The best possible ‘ΔK’* was estimated using Evanno et al. (Evanno et al. 2005) method. (1= Madinah, 2= Jeddah, 3=Makkah, 4-7= Sahil, 8= Jazan Villages, 9= Jazan Lowland, 10= Jazan Highland, 11=Najran).

**
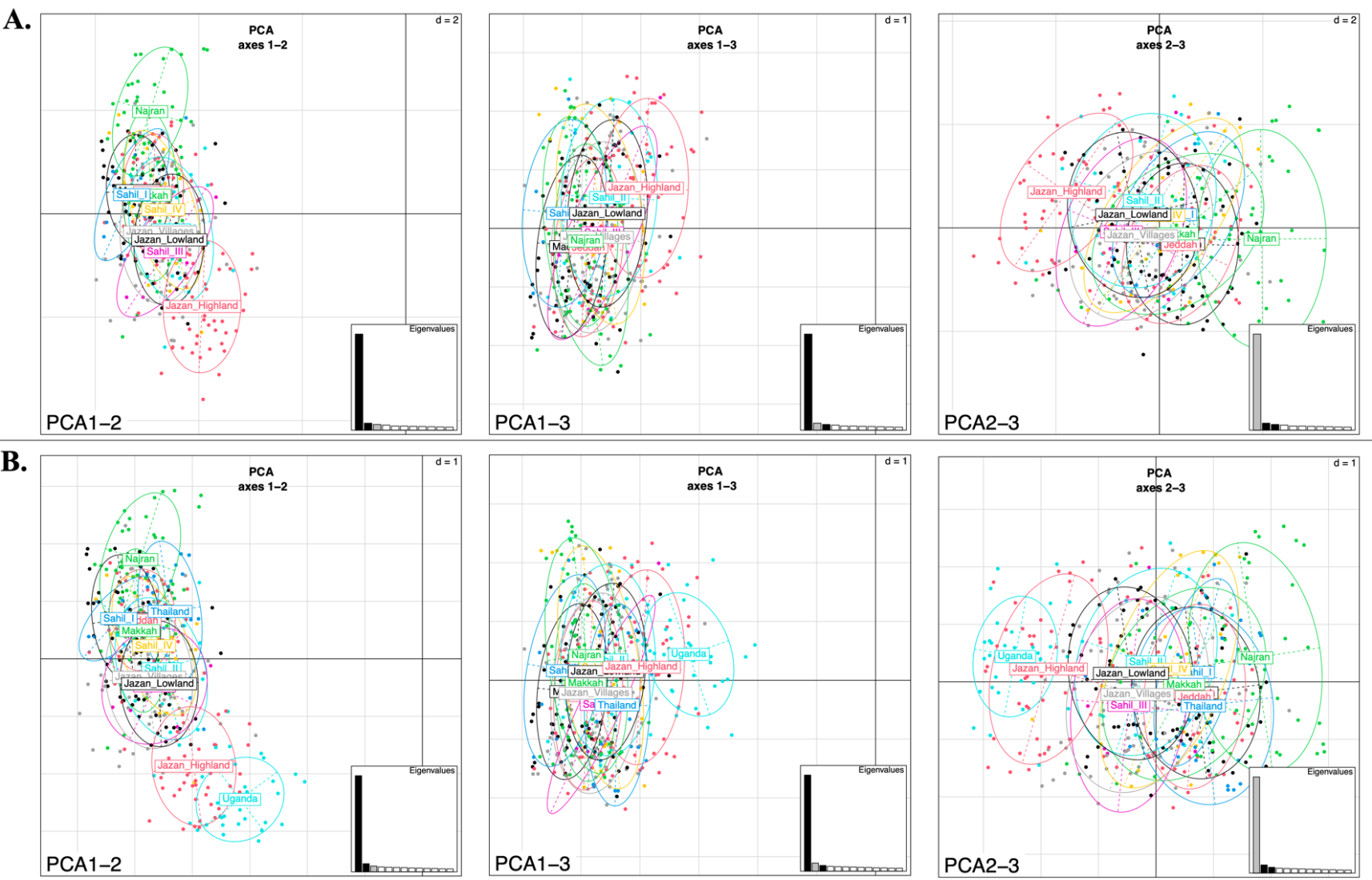
**

**Figure S4.** The Principal Components Analysis (PCA) clustering analysis for the *Ae. aegypti* populations from **A)** eleven populations; Saudi Arabia and **B)** eleven populations; Saudi Arabia, one population; Thailand, and one population; Uganda based on 17 microsatellite loci using *Adegenet* (Jombart, 2008). Each clustering colour corresponds to a population, and dots represent the individuals. A bar plot of the discriminant analysis eigenvalues corresponds to the variance ratio between groups.

#### Table S5. Priors and posteriors (logistic approach) for the ABC analysis testing scenarios on the history of Saudi Arabian *Aedes aegypti.* (Test (a) all Saudi individuals; test (b) only JAZ highland; test (c) only MAK+JED).

| Parameter | Details | Prior | Posterior |
| --- | --- | --- | --- |
| Test (a)  Admixture scenarios | Scenario 1 –  Saudi Arabia *Ae. aegypti* derived from African *(*Uganda*)*  Scenario 2–  Saudi Arabia *Ae. aegypti* derived from out-of-African *(*Thailand*)*  **Scenario 3 –**  Saudi Arabia *Ae. aegypti* derived from both African and out-of-African (Admixture) | 10 –10,000  10 –10,000  10 –10,000 | 0.0115 [0.0028, 0.0201]  0.0249 [0.0070, 0.0427]  **0.9637 [0.9430, 0.9843]** |
| Test (b)  Admixture scenarios | Scenario 1 –  JAZ highland *Ae. aegypti* derived from African *(*Uganda*)*  Scenario 2–  JAZ highland *Ae. aegypti* derived from out-of-African *(*Thailand*)*  **Scenario 3 –**  JAZ highland *Ae. aegypti* derived from both African and out-of-African (Admixture) | 10 –10,000  10 –10,000  10 –10,000 | 0.1926 [0.0000, 0.6028]  0.0033 [0.0000, 0.4108]  **0.8041 [0.7045, 0.9037]** |
| Test (c)  Admixture scenarios | Scenario 1 –  MAK+JED *Ae. aegypti* derived from African *(*Uganda*)*  **Scenario 2–**  MAK+JED *Ae. aegypti* derived from out-of-African *(*Thailand*)*  Scenario 3 –  MAK+JED *Ae. aegypti* derived from both African and out-of-African (Admixture) | 10 –10,000  10 –10,000  10 –10,000 | 0.0203 [0.0000, 0.1363]  **0.7062 [0.6575, 0.7550]**  0.2735 [0.1549, 0.3921] |

| **SCENARIO 3, test (a)** | |  | |  | |  |
| --- | --- | --- | --- | --- | --- | --- |
| **^1^Split time** | Saudi Arabia | | 10 – 10,000 | | 197 [48.1 – 459] | |
|  |  | |  | |  | |
| **Mutation rate** | Microsatellite –Stepwise Mutation Model (SMM) | | 9x10^-06^-1x10^-05^ | | 1.63 x10^-04^  [1.08 x10^-04^ – 2.79 x10^-04^] | |
|  |  | |  | |  | |

^1^ Time in generations (10 generations / year).

JAZ: Jazan

MAK+JED: Makkah + Jeddah
